# Potential associations between *Schistosoma mansoni* infection and physico-chemical characteristics and water-related human activities in Côte d’Ivoire: a cross-sectional study

**DOI:** 10.1186/s13071-024-06466-4

**Published:** 2024-10-08

**Authors:** Jean-Baptiste K. Sékré, Nana R. Diakité, Rufin K. Assaré, Jules N. Kouadio, Gaoussou Coulibaly, Cyrille K. Konan, Alain-Claver Kouamin, Aboulaye Méité, Jan Hattendorf, Mamadou Ouattara, Jürg Utzinger, Eliézer K. N’Goran

**Affiliations:** 1 Unité de Formation et de Recherche Biosciences, Université Félix Houphouët‑Boigny, 22 BP 582, Abidjan 22, Côte d’Ivoire; 2 Centre Suisse de Recherches Scientifiques en Côte d’Ivoire, 01 BP 1303, Abidjan 01, Côte d’Ivoire; 3 Programme National de Lutte Contre les Maladies Tropicales Négligées à Chimiothérapie Préventive, Ministère de la Santé et de l’Hygiène Publique, 06 BP 6394, Abidjan 06, Côte d’Ivoire; 4 Swiss Tropical and Public Health Institute, Kreuzstrasse 2, CH-4123 Allschwil, Switzerland; 5 University of Basel, P. O. Box, CH‑4001 Basel, Switzerland

**Keywords:** Côte d’Ivoire, Physico-chemical water characteristics, Risk factors, *Schistosoma mansoni*, Schistosomiasis, Water contact activities

## Abstract

**Background:**

Schistosomiasis remains a public health problem, particularly in sub-Saharan Africa. The disease is intimately connected to poverty and environmental factors. Our research was readily embedded into a multi-country schistosomiasis oversampling study. The aim of the study presented here was to determine the prevalence of *Schistosoma mansoni* and to investigate the role of water body characteristics and water-related human activities in disease transmission.

**Methods:**

In August and September 2022, a cross-sectional study was conducted in the western part of Côte d’Ivoire. Stool and urine samples were collected from 1602 and 1729 children aged 5–14 years, respectively, in 65 villages in the health districts of Biankouma, Ouaninou and Touba. Additionally, data were collected from direct observation of water-related activities at water bodies and interviews conducted with community leaders and health workers. The prevalence and risk factors for *Schistosoma* infection were assessed using generalised estimating equation models.

**Results:**

The prevalence of*S. mansoni* and *S. haematobium* were 27.4% (95% confidence interval [CI] 21.5–34.3%) and 0.1% (95% CI 0.03–0.5%), respectively. Low prevalence of soil-transmitted helminths was observed with 2.4%, 0.4% and 0.2% for hookworm, *Trichuris trichiura* and *Ascaris lumbricoides*, respectively. At the health district level, we found *S. mansoni* prevalence of 34.4% (95% CI 25.0–45.3%), 34.3% (95% CI 24.0–46.2%) and 16.3% (95% CI 9.5–26.6%) for Biankouma, Ouaninou and Touba, respectively. Female and male participants were at a similar risk of infection (29.0% vs. 26.0%, odds ratio [OR]: 1.18, 95% CI 0.92–1.50). Children aged 9–14 years showed a higher prevalence than their younger counterparts aged 5–8 years (34.5% vs. 22.7%, OR: 1.80, 95% CI 1.42–2.27). High infection prevalence was observed in villages where children were washing clothes and dishes at open surface water sites and pursued recreational activities (e.g. swimming and playing in the water). The temperature, total dissolved solids and pH of water samples showed no significant association with *S. mansoni* infection at the village unit.

**Conclusions:**

Human water-related activities such as washing clothes and playing in the water are risk factors for *S.* *mansoni* transmission. Hence, preventive chemotherapy should be combined with information, education and communication to avoid or reduce the frequency of water exposure in children as part of a comprehensive package of interventions towards elimination of schistosomiasis as a public health problem.

**Graphical Abstract:**

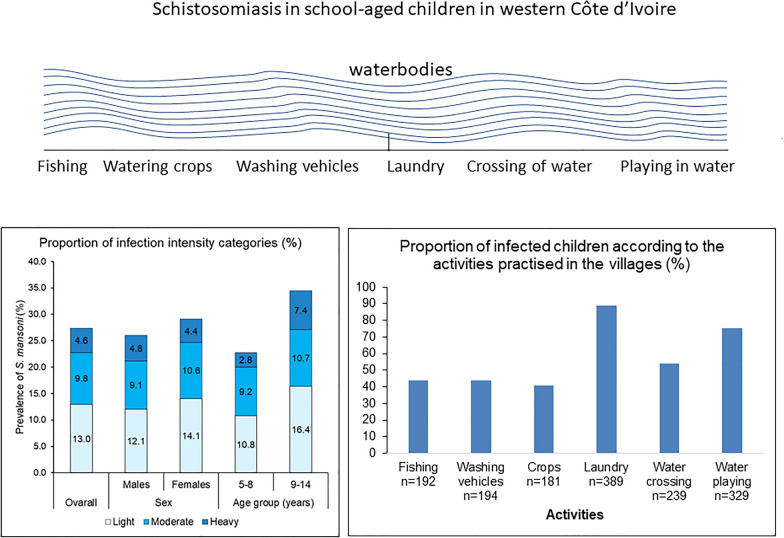

## Background

Schistosomiasis is a chronic water-based disease caused by parasitic trematode worms of the genus *Schistosoma* [1]. Six species are responsible for schistosomiasis in humans, and the three most important are *Schistosoma haematobium*, *S. japonicum* and *S. mansoni* [2]. *Schistosoma haematobium* causes urogenital schistosomiasis, while *S.* *japonicum* and *S. mansoni* are responsible for intestinal schistosomiasis [3]. The most common symptoms are diarrhoea, abdominal pain, bloody stools or haematuria, which in the long term might lead to bladder cancer and infertility [1]. It is estimated that more than 250 million people are infected worldwide, including 192 million in sub-Saharan Africa [4, 5]. In Côte d’Ivoire, a Bayesian modelling study estimated that 8.9% of school-aged children are affected by schistosomiasis [6].

According to the World Health Organization (WHO), the cornerstone for combating schistosomiasis is preventive chemotherapy with praziquantel. The aim of this strategy is primarily to reduce morbidity, while in the longer term, it might interrupt transmission when it is combined with other interventions [7, 8]. Over the past 15–20 years, preventive chemotherapy has played a key role in reducing the prevalence of *Schistosoma* infection across much of sub-Saharan Africa [8]. However, schistosomiasis remains endemic in several countries, particularly in communities without access to clean water and adequate sanitation [7, 9]. The life cycle of schistosomes requires the presence of freshwater for the parasite eggs to hatch. Furthermore, the reproductive phase of the miracidium into cercariae requires the presence of specific intermediate host snails, whose survival depends on environmental conditions [10]. Infection occurs when people come into contact with contaminated water because cercariae can penetrate the intact human skin [11]. Hence, schistosomiasis is closely linked to behavioural habits such as laundry and dishwashing, swimming and fishing [12].

The new WHO roadmap for neglected tropical diseases aims to eliminate schistosomiasis as a public health problem by 2030 [13]. To achieve this goal, it is important to understand the main causes of schistosomiasis transmission in specific social-ecological settings and take concrete measures to address them. The aim of the study presented here was to determine the prevalence of *S. mansoni* in school-aged children and to assess the underlying risk factors with a particular focus on the characteristics of water contact points and water-related activities.

## Methods

### Ethical considerations

The study obtained ethical approval from the “Comité National d’Ethique des Sciences de la Vie et de la Santé (CNESVS)” of Côte d’Ivoire (reference no. 059–22/MSHPCMU/CNESVS-kp; issued on 10 May 2022). Before any field activities, the heads of medical services and local administrative authorities were informed about the objectives and procedures of the study. The village leaders and community health workers (CHWs) were also informed, who then informed all household heads. Data were kept confidential by using participants’ individual codes instead of their names. Participation was voluntary; hence, children could withdraw at any time without further obligations. After the survey, all children aged 5–14 years in the whole study area were treated, free of charge, with praziquantel (against schistosomiasis) and albendazole (against soil-transmitted helminthiasis) according to national guidelines [14].

### Study area and participants

**Fig. 1:**
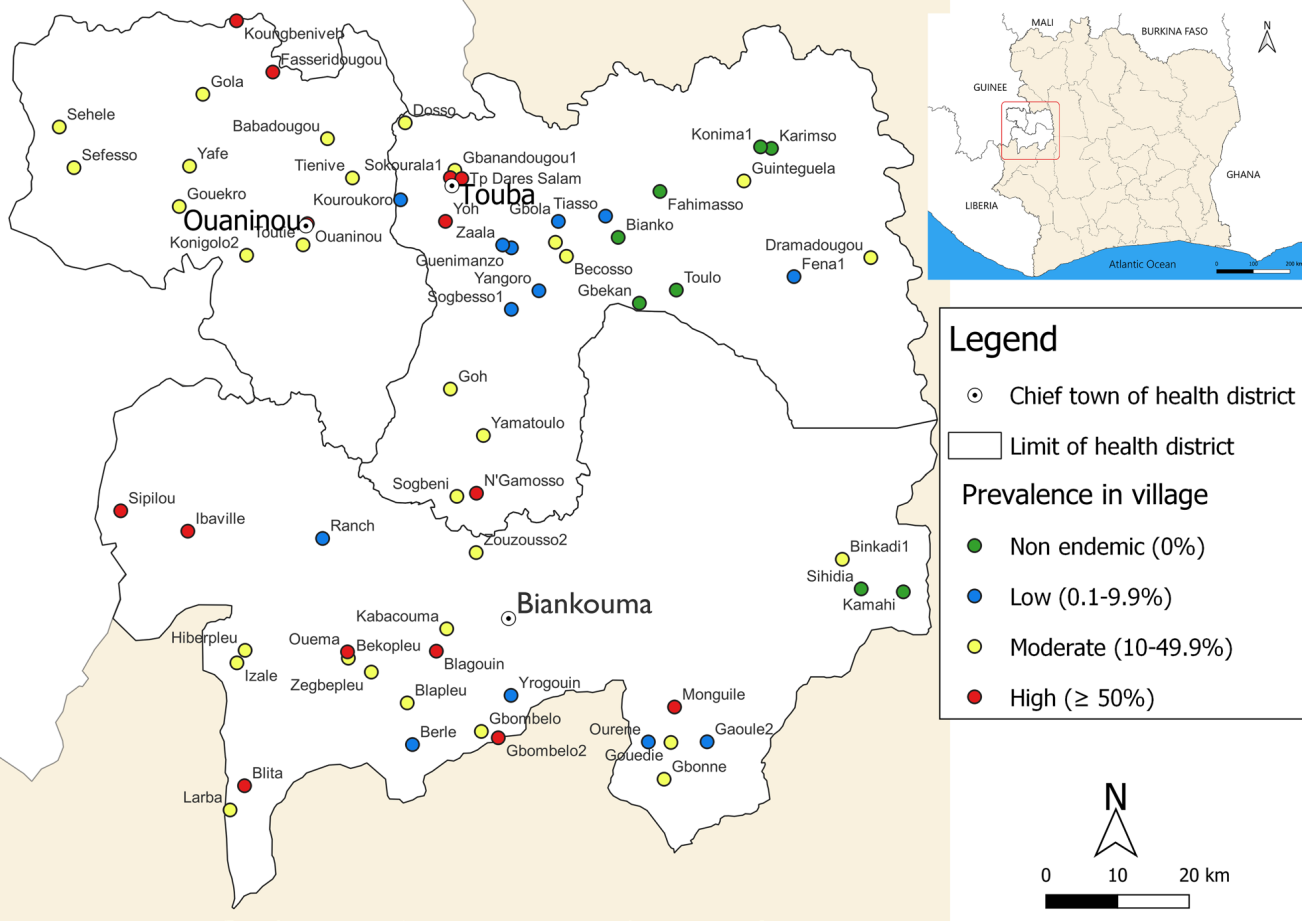
Map of the study area in the western part of Côte d’Ivoire with the estimated prevalence of *Schistosoma mansoni* among school-aged children in the 65 enrolled villages in August and September 2022

Our research was readily embedded into a multi-country schistosomiasis oversampling study. We designed a cross-sectional survey that was conducted in August and September 2022 in three health districts in the western part of Côte d’Ivoire, namely Biankouma, Ouaninou and Touba (Fig. 1). The climate is humid tropical with a rainy season from March to October and a dry season from November to February. Annual precipitation ranges between 1200 and 2000 mm. The main sources of income for the local population are cash crops with coffee and cocoa [15]. In recent years, cashew nut cultivation has become increasingly important, particularly in the Touba region. In rural areas, people mainly practice subsistence agriculture with rice, cassava, corn, bananas and yams as the main crops [16]. The western part of Côte d’Ivoire is known to be endemic for intestinal schistosomiasis [16–18]. The village inclusion criterion was the presence of a water body (e.g. ponds, rivers or small dams) in close proximity to the village. In addition, water bodies had to be accessible and used by the population.

Participants were selected using a compact segmental sampling approach. In short, the village was divided into segments of approximately 50 households using existing geographical boundaries such as roads and rivers. A single segment was then randomly selected in the presence of the village leadership. All households in the selected segment were numbered. One of two pre-prepared lists of numbers was randomly selected, and households matching these numbers were included in the study. In each selected household, children aged 5–14 years were invited to participate.

### Parasitological data collection

The day before sampling, a researcher accompanied by a CHW visited the selected households with permission from local health authorities and village leaders. The objectives, procedures and potential risks and benefits were explained and written informed consent was obtained from the children’s parents or legal guardians. In addition, children gave verbal assent. Children were issued two plastic containers (125 ml) and invited to collect an apricot-sized portion of fresh stool and a urine sample the next morning. The filled containers were collected, assigned unique identification numbers and transferred to nearby laboratories.

### Laboratory procedures

Stool samples were processed the same day for the diagnosis of *S. mansoni* and soil-transmitted helminths using standardised, quality-controlled methods. In brief, eggs of *S. mansoni* were identified under a microscope, using the Kato-Katz thick smear method [19]. Each stool sample was subjected to duplicate Kato-Katz thick smears using standard 41.7-mg templates [20]. After a clearing time of 30–45 min, the thick smears were examined by one of two experienced laboratory technicians. Urine samples were examined using a filtration method [21]. Briefly, 10 ml of urine was filtered through a polyamide filter (Nytrel) with a mesh size of 20 μm and stained with a drop of Lugol’s solution on a microscope slide. The slides were examined under a microscope by experienced laboratory technicians. Helminth eggs were counted and recorded for each species separately on summary sheets before being entered onto tablets. For quality control, 10% of the slides were randomly selected and re-examined on the same day by a third technician. In case of conflicting results, the slides were re-examined a third time and the results were discussed until agreement was reached [22].

### Questionnaire and characteristics of water bodies

A questionnaire was designed and uploaded on a tablet using an open data kit (ODK) collect application (version 2022.3.6) and specific data on water body characteristics were collected. In each village, community leaders (e.g. youth president and CHWs) who had been invited to the house of the village chief for the study were interviewed. The questionnaire was administered in French. The questions pertained to the presence of water bodies, how often people expose themselves in these water bodies and what kind of activities are pursued when getting in contact with the water. Accompanied by a local guide, the researchers visited the human-water contact sites and recorded the observed activities and determined the physico-chemical water parameter. Water samples were taken from water bodies between 10:00 and 16:00 h and the water temperature (°C), pH and total dissolved solids (TDS, ppm) were measured in situ using a multi-measurement device (Hi 98,129; Woonsocket, RI, USA).

### Statistical analysis

Data were entered directly on tablets using an ODK collect application (version 2022.3.6). The database was uploaded to a central server and extracted in Excel format. Children were classified into two age groups (i.e. 5–8 years and 9–14 years). Water contact activities were recorded and six different variables were extracted (i.e. fishing, watering crops such as vegetables and rice, washing vehicles such as bicycles, motorbikes and cars, washing clothes and dishes, crossing water bodies and playing in the water such as swimming or bathing).

Data analysis was performed using STATA version 14.2 (StataCorp LLC; College Station, TX, USA) and R version 4.3.2 (Vienna, Austria). *Schistosoma* *mansoni* infection intensity was determined by multiplying the sum of the duplicate Kato-Katz thick smear readings with a factor of 12 and expressed as eggs per gram of stool (EPG). Infection intensities were stratified into light (1–99 EPG), moderate (100–399 EPG) and heavy (≥ 400 EPG). Prevalence was classified into risk categories (low:  < 10%, moderate: 10–49.9% and high:  ≥ 50%), according to WHO guidelines [23]. Generalised estimating equation models for binary outcomes with logit link functions and independent correlation structure were applied to estimate infection prevalence and 95% confidence intervals (CIs). All models used robust variance estimators to account for potential correlation within clusters (i.e. village). Similar models were used to identify risk factors. The CIs for the geometric mean were not adjusted for clustering. It follows that the actual CIs might be slightly wider than the reported values. In all analyses, a *p*-value < 0.05 was considered statistically significant.

## Results

### Characteristics of study participants and water bodies

Overall, 1602 children from 65 villages provided stool samples. There were more males than females (855 vs. 747). In terms of age, 968 of the children (60.4%) were between 5 and 8 (mean age = 8.3; SD = 2.6) years. Urine samples were available from 1729 children, including 923 (53.4%) males.

The main activities carried out in the different villages at the water points were washing clothes or dishes, playing in water bodies (e.g. swimming, bathing, etc.) and washing vehicles (e.g. bicycles and motorbikes). Direct observations in the water bodies took place between 10:00 and 16:00 h. We observed temperatures ranging from 23.0 to 32.8 °C (mean = 26.2, SD = 1.9). The pH ranged from 2.1 to 9.9 with an average of 6.9 (SD = 1.1). The measure of TDS varied from 7 to 209 ppm (mean = 50, SD = 36).

### *Schistosoma* and soil-transmitted helminth infections

Two children were infected with *S. haematobium* owing to an overall prevalence of 0.1% (95% CI 0.03–0.5%). The prevalence of *S. mansoni* in the three health districts was 27.4% (95% CI 21.5–34.3%). Among the 65 villages, 13 (20.0%) were at low (< 10%), 30 (46.2%) at moderate (10–49.9%) and 14 (21.5%) at high endemicity (≥ 50%) (Fig. 1).

Table 1 shows the prevalence stratified by health district, sex and age group. The highest prevalences of *S. mansoni* were observed in the health districts of Biankouma (34.4%, 95% CI 25.0–45.3%) and Ouaninou (34.3%, 95% CI 24.0–46.2%). The lowest prevalence was reported in the Touba health district (16.3%; 95% CI 9.5–26.6%). At the unit of the village, the highest *S. mansoni* prevalence was 95.0%, observed in Monguilé (Biankouma health district), while the lowest prevalence (3.0%) was estimated in Guenimanzo (Touba health district). Females were at slightly higher odds of infection than males, but the difference was not statistically significant (29.0% vs. 26.0%, odds ratio [OR] = 1.18, 95% CI 0.92–1.50). Children aged 9–14 years had a higher odds of infection compared to their younger counterparts aged 5–8 years (34.5% vs. 22.7%, OR = 1.80, 95% CI 1.42–2.27) (Table 2). Soil-transmitted helminths were observed at low prevalence, 2.4% (95% CI 1.3–4.2%) for hookworm, 0.4% (95% CI 0.2–1.2%) for *Trichuris trichiura* and 0.2% (95% CI 0.1–0.6%) for *Ascaris lumbricoides*.

**Table 1:** Prevalence of *Schistosoma* and soil-transmitted helminth infection by health district, sex and age group in the western part of Côte d’Ivoire in August and September 2022

Characteristics	N children examined by Kato-Katz	*Schistosoma mansoni*		Hookworm		*Trichuris trichiura*		*Ascaris lumbricoides*		No. of children examined by urine filtration	*Schistosoma haematobium*	
		*n*	% (95% CI)	*n*	% (95% CI)	*n*	% (95% CI)	*n*	% (95% CI)		*n*	% (95% CI)
Overall	1602	439	27.4 (21.5–34.3)	38	2.4 (1.3–4.2)	7	0.4 (0.2–1.2)	3	0.2 (0.1–0.6)	1729	2	0.1 (0.03–0.5)
District												
Biankouma	729	251	34.4 (25.0–45.3)	12	1.6 (0.8–3.2)	6	0.8 (0.3–2.4)	2	0.3 (0.1–1.1)	743	1	0.1 (0.02–1.0)
Ouaninou	254	87	34.3 (24.0–46.2)	11	4.3 (1.3–13.4)	0	0.0	0	0.0	284	1	0.4 (0.1–2.4)
Touba	619	101	16.3 (9.5–26.6)	15	2.4 (0.8–6.8)	1	0.2 (0.02–1.2)	1	0.2 (0.02–1.2)	702	0	0.0
Sex												
Female	747	217	29.0 (22.4–36.7)	13	1.7 (0.9–3.4)	1	0.1 (0.02–1.0)	1	0.1 (0.02–0.9)	806	1	0.1 (0.02–0.8)
Male	855	222	26.0 (19.9–33.1)	25	2.9 (1.6–5.4)	6	0.7 (0.2–2.1)	2	0.2 (0.1–0.9)	923	1	0.1 (0.02–0.9)
Age group (years)												
5–8	968	220	22.7 (17.5–29.0)	19	2.0 (0.9–4.1)	2	0.2 (0.1–0.8)	2	0.2 (0.05–0.8)	1051	2	0.2 (0.05–0.7)
9–14	634	219	34.5 (26.6–43.4)	19	3.0 (1.6–5.7)	5	0.8 (0.3–2.2)	1	0.2 (0.02–1.1)	678	0	0.0

*n*: number of infected children

^a^Confidence interval (CI) adjusted to account for clustering

**Table 2:** Association of *Schistosoma mansoni* infection with sex and age group among school-aged children from three health districts in the western part of Côte d’Ivoire in August and September 2022

Factor	OR	95% CI^a^	*p*-value
Sex			
Male	1.00	–	–
Female	1.18	0.92–1.50	0.19
Age group (years)			
5–8	1.00	–	–
9–14	1.80	1.42–2.27	< 0.001

OR, odds ratio

^a^Confidence interval (CI) adjusted to account for clustering

**Fig. 2:**
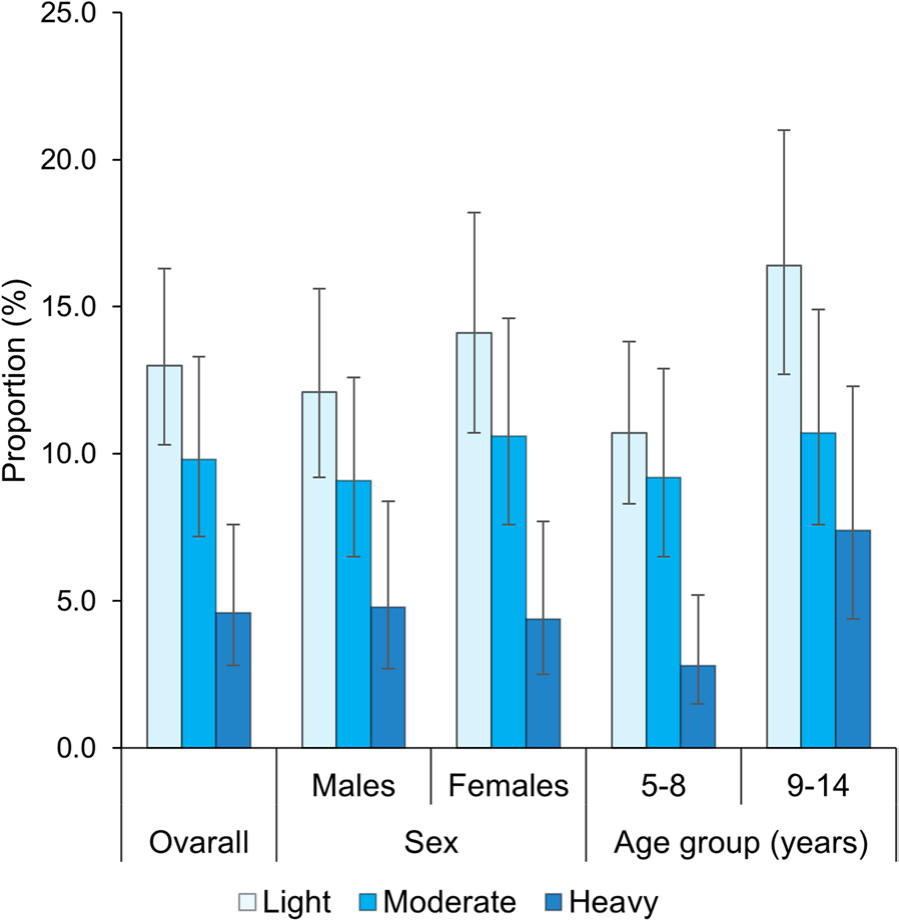
Intensity infection categories of *Schistosoma mansoni* (light, 1–99 EPG; moderate, 100–399 EPG; heavy, ≥ 400 EPG), stratified by sex and age group in school-aged children in the western part of Côte d’Ivoire in August and September 2022 (error bars represent the confidence interval of the proportion of *S*. *mansoni* infection intensity categories)

The geometric mean egg count among *S. mansoni*-positive individuals was 111.1 EPG (95% CI 98.2–125.6 EPG). The geometric mean egg count was 122.5 EPG (95% CI 104.7–143.3 EPG), 108.9 EPG (95% CI 81.5–145.7 EPG) and 85.6 EPG (95% CI 66.1–110.8 EPG) in Biankouma, Touba and Ouaninou, respectively. The corresponding estimates in males and females were 119.2 EPG (95% CI 100.0–142.1 EPG) and 103.3 EPG (95% CI 87.0–122.7 EPG), respectively. The intensity of *S. mansoni* infection was 123.5 EPG (95% CI 102.3–149.0 EPG) in children aged 9–14 years and 100.0 EPG (95% CI 85.4–117.1 EPG) in their younger counterparts aged 5–8 years. Regarding infection intensity, 9.8% of the whole study population had moderate and 4.6% heavy infection intensity (Fig. 2).

### Association of *S. mansoni* with human-water contact point parameters

**Fig. 3:**
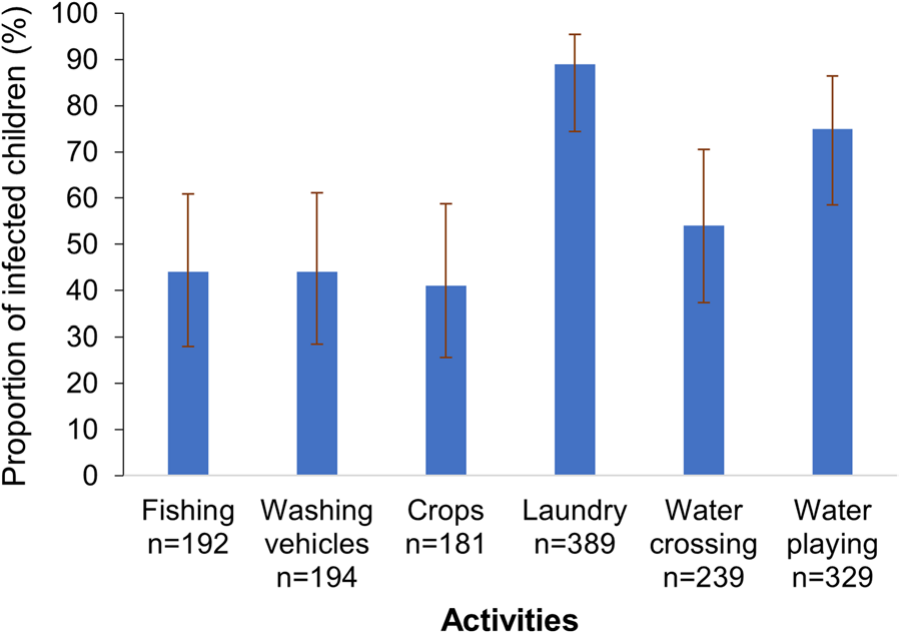
Proportion of infected children, stratified by water-contact activities in the western part of Côte d’Ivoire in August and September 2022 (error bars represent the 95% confidence intervals)

Direct observations were made at human water-contact sites to assess the potential role of water parameters and water-related activities in schistosomiasis transmission. Figure 3 shows that most of the infected children lived in villages where people used water for washing clothes and dishes (89%), followed by playing in water bodies (75%) and crossing water bodies (54%). In addition, children from villages where playing (OR = 2.57, 95% CI 0.94–6.98), fishing (OR = 1.90, 95% CI 0.95–3.79) and watering crops (OR = 1.67, 95% CI 0.84–3.26) were pursued in water bodies were at a higher odds of *S. mansoni* infection. However, the associations were not statistically significant (Table 3). We found a similar pattern for the associations with medium and high infection intensities (≥ 100 EPG). However, the odds of infection were now significantly higher in children playing in water bodies (OR: 3.17, 95% CI 1.09–9.21) (Table 3).

**Table 3:** Multivariable generalised estimating equation models to assess the association of *Schistosoma mansoni* prevalence and infection intensity with physico-chemical parameters and water-contact activities in the western part of Côte d’Ivoire in August and September 2022

Characteristics	*Schistosoma mansoni* infection (439/1602)			Medium/high *S. mansoni* infection intensity (231/1602)		
	OR	95% CI^a^	*p*-value	OR	95% CI^a^	*p*-value
Physico-chemical parameter						
pH	0.97	0.71–1.31	0.83	0.90	0.62–1.31	0.60
Temperature	1.04	0.88–1.21	0.66	1.09	0.90–1.31	0.34
TDS	1.00	0.99–1.01	0.37	1.01	0.99–1.01	0.12
Water-contact activity						
Fishing	1.90	0.95–3.79	0.07	2.12	0.83–5.36	0.11
Watering crops	1.67	0.84–3.26	0.14	1.25	0.56–2.77	0.58
Washing vehicles	0.60	0.32–1.10	0.10	0.43	0.19–0.92	0.03
Laundry	0.99	0.27–3.54	0.98	1.53	0.39–5.95	0.54
Water crossing	0.63	0.37–1.05	0.08	0.64	0.32–1.24	0.18
Water playing	2.57	0.94–6.98	0.06	3.17	1.09–9.21	0.03

OR, odds ratio

^a^Confidence interval (CI) adjusted to account for clustering

## Discussion

Over the past 10–15 years, concerted efforts have been made in Côte d’Ivoire and elsewhere in sub-Saharan Africa to combat schistosomiasis. Since 2013, settings considered at risk of schistosomiasis in Côte d’Ivoire have undergone mass treatment campaigns. Prior to this study carried out in 2022, between three and six rounds of treatments have been administered in the western part of the country. This resulted in marked declines in *Schistosoma* infection prevalence [8]. Yet, some areas are still highly endemic [24]. In the new WHO roadmap, the elimination of schistosomiasis as a public health problem is advocated. The current study is timely as it helps to determine ecological characteristics associated with schistosomiasis transmission, which is important for spatial targeting of areas at the highest risk.

Our results revealed that *S. mansoni* remains endemic in the western part of Côte d’Ivoire with only a slight decrease in the prevalence of infection among school-aged children compared to previous studies [25–27]. Among the 65 villages enrolled, 14 were highly endemic for schistosomiasis (prevalence ≥ 50%) and hence deserve special attention. In addition, several individuals had heavy-intensity infections (≥ 400 EPG). Although multiple treatment rounds were administered to control schistosomiasis in this area [28, 29], the prevalence remains high with an overall prevalence estimated at 27.4%. In other words, according to our findings, one out of four children suffer from intestinal schistosomiasis. This is partly because of the study area is characterised by numerous rivers discharging from the mountains to the plains. These water bodies are frequently used for domestic activities such as washing clothes and dishes and for recreational activities by children [12]. Additionally, rapid re-infection might occur after preventive chemotherapy, as many households lack access to clean water, improved sanitation and hygiene [30–33]. It should also be noted that preventive chemotherapy is mainly targeted to school-aged children, while other groups (e.g. preschool-aged children and adults) are excluded; thus, breaking transmission is hard to achieve [34, 35]. Indeed, we found a considerable proportion of heavy and moderate intensity infection, with respectively 4.6% and 9.8%. This observation contributes to maintaining schistosomiasis transmission in this setting, as emphasised by previous studies pertaining to reinfection with schistosomiasis [36]. The authors of a previous study found that children with light infections had significantly higher cure rates than those with moderate or heavy intensity infections [36].

The proportion of infected females was quite close to that observed in males. This finding is in agreement with previous studies on *S. mansoni* [37, 38] and *S. haematobium* [39]. Water-contact patterns might be similar by either sex. Although females and males might use water bodies for different purposes, the frequency might be similar, because when females practise domestic activities such as washing clothes and dishes, males might pursue recreational activities such as swimming [40]. In contrast, other prior studies reported a significantly higher prevalence in males [26, 29, 41–43]. Some authors speculated that males were generally freer and indulged in swimming, while females, who were more closely supervised by their parents and guardians, often stayed at home to help with household chores [26]. In terms of age, we found that 9- to 14-year-old children had higher odds of infection than their younger counterparts. This observation is in line with a study from Egypt [44]. A likely explanation for this observation is that children aged 9–14 years are freer to go and play anywhere, especially in water bodies. This is not the case for younger children, who are more closely supervised by their parents and guardians. Schistosomiasis is known to be a disease strongly linked to human behaviour, particularly the frequenting of water bodies [45]. Our study found no statistically significant relationship between *S. mansoni* in school-aged children and water-related activities. However, the findings showed that many children were infected in villages where people wash clothes and dishes and play in water bodies. This observation could be explained by the fact that washing and playing in water, such as swimming, prolongs the duration of contact with schistosome-infested water [46]. Moreover, during swimming, a favourite water activity for children, they expose their whole body to water, resulting in more contact with infested water [41]. Similar findings were reported elsewhere [47]. Another explanation might be the fact that, during playing, children release stool and urine into the water. This helps to perpetuate the schistosome life cycle and hence maintain the transmission of *Schistosoma* infection. However, the lack of significance might be because the same activities are practised in several villages, which might reduce the variability of the model parameters.

Regarding the relation between *S. mansoni* and the physico-chemical parameters of water bodies, we did not find any statistical significance between *S. mansoni* and any of the parameters investigated. Of note, *Biomphalaria pfeifferi* is the intermediate host of *S. mansoni* in Côte d’Ivoire [18]. Additionally, schistosomiasis distribution is focal, with transmission occurring only in areas where snail intermediate hosts are present and where human populations frequently come into contact with infested water [48]. Hence, any factor favouring the proliferation of intermediate hosts in schistosomiasis endemic areas could exacerbate the transmission. Although favourable water conditions might stimulate attachment and skin penetration by cercaria [49, 50], studies have shown that pH has no significant influence on the growth rate and fecundity of *B. pfeifferi* or snail abundance [51, 52]. This assertion was in line with a lack of an association between water parameters and *S. mansoni*. However, a previous study assessing the effect of increasing temperature on *S. mansoni* demonstrated that infection risk increases sharply when temperatures increase above a minimum threshold that is necessary for sustained transmission [10]. Other studies showed that the abundance, survival and distribution of intermediate host snails, as well as their parasites, may be strongly influenced by temperature [53, 54]. Moreover, using negative binomial regression, a recent study conducted in Egypt has shown that TDS is associated with the abundance of *Biomphalaria alexandrina*, another intermediate host of *S. mansoni* [44].

Our study has several limitations. First, we did not carry out a malacological survey, which might provide definitive explanations of risk factors on the transmission of *S. mansoni* infection. Second, investigations were carried out in only 65 villages, with one human-water-contact site per village. This relatively small number of investigated human-water-contact sites could affect the statistical power; hence, care is needed when drawing final conclusions. Third, our study pursued a cross-sectional design, which does not consider the seasonal variation of water parameters. Moreover, we made measurements at only one time point of the day, excluding daily variation.

## Conclusions

Our study revealed that intestinal schistosomiasis is still endemic in the western part of Côte d’Ivoire, with a moderate level. Certain human behaviours like washing clothes and dishes and playing in water bodies seem to be linked to *S. mansoni* transmission, though no significant association was found. The same is true for water parameters, temperature, TDS and pH. A malacological survey might provide a complementary understanding of these factors in the transmission of *S. mansoni*. For an effective control of schistosomiasis, drug administration should go hand in hand with information, education and communication of the affected communities to induce a change in behaviour and improve access to clean water. Larger and more in-depth studies, complemented with malacological surveys, could enhance the understanding of the links explored in this study.

## Data Availability

The data supporting the findings of the study must be available within the article.

## Abbreviations

CHW: Community health worker
CI: Confidence interval
CNESVS: Comité National d’Ethique des Sciences de la Vie et de la Santé
EPG: Eggs per gram of stool
OR: Odds ratio
SD: Standard deviation
TDS: Total dissolved solids
WHO: World Health Organization
